# The Influence of Electricity on Protoplasm

**Published:** 1890-11

**Authors:** 


### THE INFLUENCE OF ELECTRICITY ON PROTOPLASM.

THE *American Microscopical Journal*, for August, 1890, contains the address of Dr. Fell, of this city, before the American Society of Microscopists, on the above subject. In it are found the results of microscopical examination of some of the tissues of a calf killed by electricity. The examination of the heart muscle, made by Professor S. H. Gage, of Cornell University, is as follows : In examining the calf-heart muscle sent me, I made sections, stained and mounted in balsam. As a criterion to guide me, the heart of a calf, butchered in the ordinary way, was obtained, and preparations were taken from the same part of the heart of the two specimens and treated exactly alike. After comparing parallel preparations, no constant differences could be found. In the heart killed by electricity, the longitudinal striation of the muscle-cells was very clear, the fibrillæ seeming to be separated by a comparatively white, wide, clear line, and in the fibrillæ, the dark band was very marked, giving the appearance of a row of light and dark tubes. From the width of the inter-fibrillar light line the transverse striation was not so marked as the longitudinal. Later, the distinction broke down as similar, if not quite so marked, in the heart muscle of the butchered calf. The nuclei of the muscle-cells were scrutinized with the greatest care, but no difference could be discovered.

The result of the microscopical examination of the brain, made by Dr. Wm. C. Krauss, of Buffalo, N. Y., was as follows: The pia was non-adherent, pial vessels were somewhat injected, the brain substance was firm and resistant, not brittle, and no petechial extravasations were discovered on section. A slight discoloration of the pia and underlying brain substance, over the frontal and occipital lobes, was no doubt produced by the thermic action of the electric current.

The sections took on an indistinct diffused stain. The ganglion cells lacked that sharpness and clearness of outline generally found in normal brain tissues. As to the physical condition of the ganglion cells, there appeared to be no material change; nucleus cell, body and poles were in normal condition; the same was true of the vessels and neuroglia cells. No evidence of hemorrhage into the brain tissue could be discerned.

The peri-ganglionic spaces were found free and unobstructed. The result of the microscopic examination is, therefore, negative as far as the physical condition of the separate brain elements is concerned. Whether the diffused appearance of the sections can be attributed to some chemical change in the protoplasm, is left unanswered.

